# Videoconference fatigue from a neurophysiological perspective: experimental evidence based on electroencephalography (EEG) and electrocardiography (ECG)

**DOI:** 10.1038/s41598-023-45374-y

**Published:** 2023-10-26

**Authors:** René Riedl, Kyriaki Kostoglou, Selina C. Wriessnegger, Gernot R. Müller-Putz

**Affiliations:** 1 Digital Business Institute, University of Applied Sciences Upper Austria, Campus Steyr, Steyr, Austria; 2 Institute of Business Informatics – Information Engineering, University of Linz, Altenbergerstrasse 69, 4040 Linz, Austria; 3 Institute of Neural Engineering, Graz University of Technology, Graz, Austria; 4 BioTechMed Graz, Graz, Austria

**Keywords:** Neuroscience, Psychology

## Abstract

In the recent past, many organizations and people have substituted face-to-face meetings with videoconferences. Among others, tools like Zoom, Teams, and Webex have become the “new normal” of human social interaction in many domains (e.g., business, education). However, this radical adoption and extensive use of videoconferencing tools also has a dark side, referred to as videoconference fatigue (VCF). To date only self-report evidence has shown that VCF is a serious issue. However, based on self-reports alone it is hardly possible to provide a comprehensive understanding of a cognitive phenomenon like VCF. Against this background, we examined VCF also from a neurophysiological perspective. Specifically, we collected and analyzed electroencephalography (continuous and event-related) and electrocardiography (heart rate and heart rate variability) data to investigate whether VCF can also be proven on a neurophysiological level. We conducted a laboratory experiment based on a within-subjects design (N = 35). The study context was a university lecture, which was given in a face-to-face and videoconferencing format. In essence, the neurophysiological data—together with questionnaire data that we also collected—show that 50 min videoconferencing, if compared to a face-to-face condition, results in changes in the human nervous system which, based on existing literature, can undoubtedly be interpreted as fatigue. Thus, individuals and organizations must not ignore the fatigue potential of videoconferencing. A major implication of our study is that videoconferencing should be considered as a possible complement to face-to-face interaction, but not as a substitute.

## Introduction

As a consequence of the restricted social mobility that resulted from COVID-19-induced lockdowns, videoconferencing has been adopted by many people and organizations worldwide. Systems such as Zoom, Microsoft Teams, Cisco Webex, Skype, among others, have been implemented to maintain communication in various areas, including education, business, health care, science, judiciary, and in several private domains^1–4^. Despite the fact that Skype, which was launched in 2003, has been popular as videoconferencing tool for two decades, adoption rates of most other tools astronomically increased when the first lockdowns were imposed in spring 2020. As an example, the number of *zoom.us* monthly visits was 71.6 million in December 2019, increased to 1.9 billion in April 2020, had its all-time-high with 2.8 billion in October 2020, and was at 943 million in March 2023^5^. Thus, in March 2023 the *zoom.us* monthly visits were approximately 13 times higher than just before the beginning of the COVID-19 pandemic in December 2019. In essence, videoconferencing has become a widely-used substitute for face-to-face communication in many domains. As a consequence of the use of videoconferencing tools during the COVID-19 pandemic, people and organizations have been able to maintain communication, a necessary foundation for the functioning of economies and societies. Use of videoconferencing also saves travel costs^6,7^ and helps to preserve the environment^8^.

However, this radical adoption and extensive use of videoconferencing tools also has a dark side, referred to as videoconference fatigue^9^ (hereafter VCF); note that Zoom Fatigue is used as a synonym for VCF in the literature, despite the fact that this fatigue also applies to the exhaustion that may result from the use of other videoconferencing tools (e.g., Teams, Webex, Skype)^9–11^. VCF is defined as “somatic and cognitive exhaustion that is caused by the intensive and/or inappropriate use of videoconferencing tools” (^9^, p. 157). Self-report evidence, collected all around the world, indicates that VCF is a serious issue^12–16^. The journal *Australasian Psychiatry* even published a short article in which the authors write about “a new diagnosis of paramount significance […] which may be included in international diagnostic classifications [and this] proposed diagnosis is based on clinical observations of an insidious and debilitating video-meeting mediated disorder” (^17^, p. 669). The phenomenon’s relevance is further substantiated by evidence showing that VCF comes along with increased depression and burnout tendencies^18^. Moreover, evidence indicates that—if compared to face-to-face interaction—videoconferencing inhibits the production of creative ideas^19^.

Against the background of the presented developments since 2020 and considering that it is predicted that the use of videoconferencing tools will stay high in the future, VCF already constitutes, and even so more in the future, a phenomenon of global relevance, both in science and practice. Major reasons for the predicted future relevance of VCF are ever increasing home office rates^20^ and the increasing use of videoconferencing tools in education^21^.

Analysis of the academic literature which has appeared since the emergence of the phenomenon in April 2020^22^ reveals two major aspects. First, the available scientific research focuses on the causes of VCF rather than its physiological, psychological, and behavioral consequences^9,23^; for a review of causes which distinguishes personal, organizational, technological, and environmental factors, see^24^. Second, the existing studies have shown the existence of VCF based on self-reports only. However, based on self-reports alone it is hardly possible to provide a comprehensive understanding of a cognitive phenomenon. This fact is particularly true for VCF, because evidence indicates that measurement of fatigue, exhaustion, and stress in the context of information and communication technology use should be carried out with both physiological and self-report measures^25–28^. What follows is that a significant research gap exists.

To the best of our knowledge, to date no scientific study has investigated whether videoconferencing results in fatigue on a brain level. Hence, the present study examines the following main research question: *Does videoconferencing lead to fatigue on a brain level?* In order to demonstrate potential fatigue effects, in our experimental study we measured users’ ongoing electroencephalogram (EEG) during a videoconferencing session, as well as their event-related potentials (ERPs) before and after a videoconferencing session based on a cognitive attention task (i.e., oddball paradigm). Importantly, we contrasted these results with corresponding EEG data from a face-to-face condition in which the exact same content was provided to the meeting participants. The context of our study was a 50-min university lecture. Moreover, we complemented the brain data with electrocardiography (ECG) data. Specifically, we investigated heart rate (HR) and heart rate variability (HRV). Finally, we also measured fatigue and mood based on self-report instruments. In addition to fatigue, we added mood as outcome variable in our study because evidence indicates that increased fatigue typically worsens mood^29^.

Our study contributes in novel ways to the academic literature. First, the current study is the first which provides EEG evidence showing that a 50-min lecture administered via a videoconferencing tool leads to fatigue on a brain level. Second, this brain data is complemented by autonomic nervous system data showing also notable changes in HR and HRV which signify physiological fatigue^30–32^. Therefore, the present study complements the existing self-report studies, which have demonstrated the existence of VCF. Third, our study also has a major implication for practice, namely that videoconferencing should be considered as a complement to face-to-face interaction, but not as a substitute.

Next, we continue with a description of the neurophysiological correlates of fatigue. Afterwards, we describe our methods and materials, followed by a detailed presentation of the results and their discussion. We also outline limitations and potential avenues for future research, and we close this paper with a concluding statement.

## Neurophysiological correlates of fatigue

In order to establish the possible fatigue potential of videoconferencing on a brain level, sensitive brain fatigue indicators are needed. In our study, we applied fatigue indicators related to both the continuous EEG and ERPs. In addition, based on ECG data we also analyzed HR and specific HRV measures to detect possible fatigue effects of videoconferencing.

### Fatigue detection based on EEG

#### Continuous EEG

The continuous EEG is the measurable part of brain activity that permanently takes place; it is basically the measurement of electrical signals in a specific time window and hence is unrelated to specific short-term stimuli or events (e.g., all the specific events which take place during a videoconferencing session). In the healthy waking brain, a large portion of the signal power originates from rhythmic oscillations in a frequency bandwidth from 5 Hz (or sometimes less) up to 25 Hz (in case of high arousal states the oscillations even increase to frequencies of 200 Hz)^33–35^. Depending on the specific research question, scholars typically analyze frequency information in the Delta (0–4 Hz), Theta (4–8 Hz), Alpha (8–13 Hz), Beta (13–30 Hz), and Gamma (30–200 Hz) bands based on specific time windows and electrode locations (frontal, central, parietal, and occipital locations) (the exact limits of these frequency bands vary slightly in the scientific literature^34^). EEG frequency bands are related to mental states such as fatigue^33^.

In general, while very low frequency indicates deep sleep or even coma (i.e., Delta), higher frequencies indicate more aroused brain states (e.g., Beta is typically related to active concentration and focused attention and Gamma to pronounced arousal states) (for a review, see^36^). The frequency bands in between (i.e., Theta and Alpha) typically indicate states in which the brain is neither focused nor aroused, but awake^36^. As a result, when people are actively engaged in a mental task (like a videoconferencing session), the progressive rise in Theta^37–40^ and Alpha activity^38,41^ over time serves as an indicator of brain fatigue. Against this background, it is not surprising that^42^, for example, indicate that changes in Alpha and Theta power are a “reliable indicator of fatigue” (p. 119). In the same vein,^43^ indicate that Theta rhythm “is related to brain fatigue and has a sensitive response to fatigue” and Alpha rhythm “is considered to be the most sensitive indicator of brain fatigue” (pp. 5–6). Another study by^44^ showed a band power increase in Theta and Alpha frequency bands with increasing experiment duration, supported by subjective ratings and behavioral measures. Against this state of knowledge, we calculated the power spectral density and compared them across defined time windows to detect changes in the Theta and Alpha bands, thereby revealing possible fatigue effects on a brain level.

Moreover, it is well-established in EEG research to extract ratios between different frequency bands in order to determine mental states^36^. Regarding the relationship between such ratios and fatigue, research indicates that attention deficit and impaired attention control, if conceptualized as a consequence of mental fatigue^45,46^, is related to the spectral power ratio of *Theta*_*frontal*_/*Beta*_*frontal*_^47–49^. This finding is substantiated by evidence showing that increased levels of *Theta*_*frontal*_*/Beta*_*frontal*_ have also been detected during mind-wandering episodes compared to episodes of focused attention^49^. Thus, this ratio can be used as indicator for mental fatigue.

#### Event-related potentials (ERPs)

The ERP describes a waveform complex resulting from perception of an external stimulus, and an ERP typically refers to averaged EEG responses that are time-locked to the stimulus^50^. It follows that, in ERP research, a participant is repeatedly exposed to the same experimental stimulus or stimuli (if more than one is used). In general, ERPs reflect transient, fixed latency, and fixed polarity-evoked responses to a certain stimulus^36^. Examples of stimuli are the presentation of words, sounds, or pictures. ERP waves comprise a series of positive and negative voltage deflections related to several underlying perceptual, motor, and cognitive components, and components are referred to by a letter (P or N) that indicates polarity (positive or negative) and by a number that indicates either the latency in milliseconds (e.g., 100, 200, or 300 ms) or the component’s ordinal position in the waveform (e.g., 1st, 2nd, or 3rd). Moreover, different ERPs are related to different scalp distributions^36^.

A prominent component is the P300, which occurs in the latency range around 300 ms and is usually provoked by the so-called oddball paradigm^51^, a specific experimental design consisting of the presentation of sequences of a repetitive target stimulus, infrequently interrupted by a deviant stimulus. Evidence indicates that the P300 is larger after a target stimulus is detected (in our experiment, for example, pictures of faces) if compared to other stimuli (in our experiment, for example, pictures of landscapes; details are provided in the Methods and Materials section). The P300 occurs if a participant is actively engaged in the task of detecting the targets and it is a sensitive measure of cognitive attention^42,52–55^. Specifically, evidence indicates that if cognitive attention becomes diminished as a consequence of mental fatigue, a reduced P300 amplitude can be observed^56^. Studying the fatigue-related aftereffects of human interaction with computers,^57^ found that the P300 amplitude was smaller after human–computer interaction tasks if compared to paper/pencil conditions, and they indicate that reduced P300 amplitudes “can be interpreted as a sign of fatigue or depletion of resources” (p. 654). Other components—specifically the P3a and N2—have also been shown to be correlated with fatigue and closely related constructs such as attention^58–62^. In order to demonstrate fatigue effects on a brain level in our experiment, we therefore also applied an oddball paradigm before and after the two experimental sessions (videoconference and face-to-face conditions) and looked for potential changes in the P300, P3a, and N2.

### Fatigue detection based on ECG

In addition to EEG measures, we also recorded ECG data, analyzed HR data, and applied specific HRV measures to detect possible fatigue effects of videoconferencing.

#### Heart rate (HR)

HR is an important indicator of an individual’s physiological state, and it is also related to mental states such as arousal or fatigue, among others; HR is widely used to measure autonomic nervous systems (ANS) activity^63^. The sympathetic part of the ANS is stimulatory and hence its main function is the implementation of a “fight-or-flight” response in stressful situations^64^. Such a response triggers a number of physiological processes, one of which is heartbeat acceleration. What follows is that in situations of arousal HR increases, a fact which has also been confirmed in arousing human–computer interaction situations (for a review, see^65^). This increase in HR along with several other physiological responses (e.g., increase in blood pressure and skin conductance elevation) prepares the organism in order to secure optimal performance and functioning of the body. However, prolonged physical and/or mental activation that results from sympathetic activity usually—after some time—leads to activation of the parasympathetic branch of the ANS, which is inhibitory, thereby reducing arousal and stress (Kolb & Whishaw, 2009). Against the background of this regulative mechanism, what follows is that fatigue is correlated with reduced HR (due to parasympathetic activity). Recent empirical evidence confirms that mental fatigue is correlated with decreased HR^32,66^.

#### Heart rate variability (HRV)

HRV refers to the temporal variations between successive heartbeats and has been commonly used to characterize the balance between the sympathetic and parasympathetic branches of the ANS^67^ and to assess the ANS adaptability and responsiveness to external influences such as stress, emotions, cognitive phenomena, and several other environmental factors^67^. HRV data are described as an important complement to brain data in the study of the physiological correlates of affective and cognitive phenomena^68^. Research has also established a significant relationship between mental fatigue and HRV^32^. Physiologically, such a relationship is highly plausible as a major function of the ANS is adaptive modulation of bodily processes, including cognition and subsequent behavior, under changing environmental influences^63,69^. Modulation of the heartbeat, and hence HRV, is significantly driven by the sympathetic (arousal) and parasympathetic (relaxation) branches of the ANS. Based on a review of related literature^70–72,32^ concluded that “HRV is assumed to be an ideal tool to examine the association between fatigue-vulnerable psychological operations and autonomic processes. Previous studies examining the HRV-fatigue associations indeed converged on the conclusion that HRV is a significant associate of fatigue” (p. 2).

Specifically, we used the following HRV measures to identify possible fatigue effects: pNN50, RMSSD, SDRR, LF_nu_, HF_nu_ and ln[LF/HF] (details on these measures are provided in the Methods and Materials section). We selected these measures because they are identified as important measures in the current literature on the association between mental fatigue and HRV^30,31^. In essence,^32^ indicate that “the parasympathetic branch of the autonomous nervous system functioning as a relaxation system tends to be activated under increasing mental fatigue” (p. 1). What follows is that VCF is correlated with increased HRV.

## Methods and materials

The experiment was conducted based on the Declaration of Helsinki and was approved by the responsible ethics committee at Graz University of Technology, Austria.

### Participants

Thirty-five people (N = 35) voluntarily participated in this within-subjects experiment. This sample size constitutes the typical sample size of EEG experiments on neurophysiological consequences of human interaction with, or via, information and communication technology (^73^, p. 25) and of applied neuroscience studies in general^74^. Participants were almost gender balanced (male: 20, female: 15) and their mean age was 24.06 years (SD 2.04, range 20–29). There was no significant age difference in the groups of females and males (*p* = 0.3607 based on the Wilcoxon rank-sum test). As a precondition for taking part in the experiment (to avoid possible influences on fatigue), participants had to confirm that they at least have five hours of sleep in a typical night (M = 7.4 h, SD = 1.2). All participants confirmed that they have normal sleeping behavior, both in general and the night before the experimental sessions. All participants gave written informed consent before participation.

### Experimental procedure

The subjects, all of which were enrolled students, participated in a 50-min lecture at a public university in Austria (engineering topic) and the lecture was given in both conditions (videoconferencing, face-to-face) by the same male university professor. The lecture (either videoconferencing or face-to-face) was given on 4 days in a week (one lecture per day), the same 4 days the week after, to always 7 to 10 participants in a classroom setting. The exact numbers of students were in the first week and second week as follows: W1—day 1 (9), day 2 (7), day 3 (9), day 4 (9); W2—day 1 (9), day 2 (10), day 3 (9), day 4 (8) (note that one subject did not show up in the first week and hence participated in both conditions in the second week). In order to avoid confounding effects caused by the possible interaction between the lecturer and students, all participants were informed that no interaction is possible with the professor during the lecture and this information was provided in both conditions. The videoconferencing session was administered as a pre-recorded video. In the debriefing phase all participants indicated that they did not notice that the videoconferencing session was actually not a live-lecture. The participants’ task was to listen to the presentation by the lecturer. The participants were informed that after the lecture a performance check will be conducted based on a short questionnaire; the purpose of administering this check was to ensure the participants’ engagement. Note that in this check we deliberately only included simple questions as our goal of the announcement of the performance check was to keep the concentration of the participants high (to ensure engagement), and *not* to create significant variance in performance data across the participants and to thereby make it possible to add these data as an outcome variable to our experiment.

To avoid possible carry-over effects in our within-subjects experiment, approximately half of the subjects (N = 18, male: 10, female: 8, mean age: 23.94) first participated in the videoconferencing and then a week later in the face-to-face condition, while it was the opposite for the rest of the subjects (N = 17, male: 10, female: 7, mean age: 24.17). Assignment to the starting condition was random. Participants were not informed about the goal of the study (i.e., whether videoconferencing leads to fatigue). However, in the debriefing phase at the end of the experiment full information about the study purpose was provided to all participants.

### Study protocol

Each session was structured to include a 15-min introduction phase, which encompassed greetings, instrument setup, task instructions, Brief Mood Introspection Scale (BMIS) and demographics questions, and a relaxation phase. This was followed by a 5-min oddball paradigm, and then the 50-min lecture. Once the lecture was completed another 5-min oddball paradigm followed, and then the participants were instructed to respond to the BMIS and the Zoom Exhaustion and Fatigue (ZEF) questionnaires, as well as to three questions pertaining to the lecture topic (engagement check). The session was concluded by removing the instruments and debriefing the participants.

**Figure 1:**
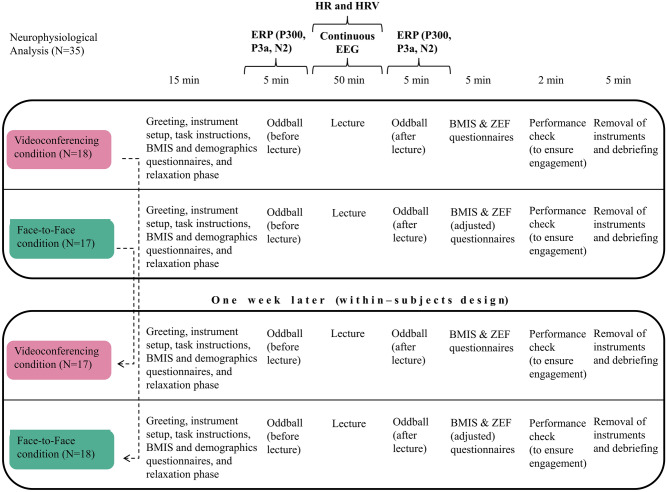
Study Protocol. A total of 35 subjects participated in the study, divided into two groups (within-subjects design). One group attended the videoconference lecture first and then the face-to-face lecture a week later, while the other group did the opposite. The protocol included a greeting session with instrument setup, task instructions and self-report items, an oddball paradigm before and after the lecture, and the lecture itself. Participants were asked to complete the Brief Mood Introspection Scale (BMIS) questionnaire before and after the lecture and the Zoom Exhaustion and Fatigue (ZEF) after the lecture (ZEF adjusted in the face-to-face condition). Moreover, a performance check with three questions was included to secure participants’ engagement.

To assess the participant’s mood, we utilized BMIS^75^, administered before the first oddball and after the second oddball task. Specifically, the participants were asked to evaluate their current emotional state by rating from 1 to 4 (with 1 being low and 4 being high) the degree to which sixteen different adjectives described their mood (e.g., happy, tired, gloomy, active). The BMIS score was obtained by subtracting the total score of the negative mood adjectives (referred to as negative score) from that of the positive/pleasant ones (referred to as positive score). Furthermore, different forms of fatigue were evaluated using the Zoom Exhaustion and Fatigue (ZEF) scale^11^. Herein, we used a validated German version of the ZEF scale^18^. The ZEF scale contains eighteen questions designed to measure visual, social, motivation, and general fatigue (rated from 1 to 5, with 5 indicating higher levels of fatigue). To calculate an overall ZEF score, the ratings of all fatigue items were averaged. The ZEF scale was administered after the lecture in both the face-to-face (adjusted version) and videoconferencing conditions. Details regarding both questionnaires can be found in the Supplementary Material. Figure 1 graphically summarizes the study protocol.

### Devices and measurement

EEG and ECG was measured using five 16 channel g.USBamp biosignal amplifiers (g.tec. medical engineering GmbH, Austria) at a sampling rate of 512 Hz. For the EEG, we used 5 passive Ag/AgCl electrodes placed at Fz, Cz, Pz, O1 and O2. Reference was placed on Fpz and ground on the left mastoid. We concentrated solely on the midline channels (Fz, Cz, Pz) given that the P300 response exhibits a more prominent distribution across central and parietal brain regions along this axis^54,76,77^. Additionally, we prioritized the observation of general activity patterns and network dynamics that might span both left and right hemispheres. We also aimed to concurrently record data from as many individuals as possible using the limited number of amplifiers at our disposal, thus mitigating prolonged waiting times during EEG cap setup and placement. O1 and O2 were chosen to investigate the laterality of the visual field. ECG was monitored using a 2-lead configuration, whereby one electrode was placed under the right clavicle near the right shoulder and the other under the left clavicle along the mid-clavicular line within the rib cage. The left mastoid ground electrode was used as a reference for the ECG measurements. Two participants were measured using one amplifier, ensuring electrical isolation for the ground connection within each participant. Measurement of the two participants per amplifier was carried out in synchrony, started manually. The analysis of the data was performed in Matlab, Mathworks.

With respect to continuous EEG measurement, the data was first high-pass filtered, using zero-phase, windowed-Sinc FIR filtering (based on a Hamming window; see also *pop_eegfiltnew.m* function from EEGLAB) with a cut-off frequency of 0.5 Hz and a filter order of 1128, to remove low frequency drifts. Note that the transient response of the filter was attenuated by the time the experiment began. Power line noise interference at 50 Hz and harmonics was suppressed using a comb filter and eye-blink artifacts were removed using wavelet enhanced independent component analysis (wICA)^78^, selecting components through visual inspection. Unlike the conventional ICA algorithm, which completely zeroes out the chosen components, the enhanced wavelet ICA method employs wavelet transformation on these components. Any wavelet coefficients that exceed a predetermined threshold are subsequently set to zero. Although we primarily adhered to the threshold recommended by^78^, occasional adjustments were necessary. We, then, computed the band power in the Delta (D, 0.5–3.5 Hz), Theta (T, 4–7.5 Hz), Alpha (A, 8–12.5 Hz), and Beta (B, 13–30 Hz) bands based on non-overlapping windows of 3 min in the frontal (Fz), central (Cz), parietal (Pz), left occipital (O1), and right occipital (O2) areas. The power in each band was standardized by dividing it by the total signal power. We specifically opted for a 3-min window duration to ensure stable estimates, thereby reducing the impact of high frequency noise and short-term fluctuations. Furthermore, this choice highlights slow spectral changes that manifest during lectures. The power spectral density was estimated using Welch’s method. Specifically, the signals were divided into eight segments with 50% overlap. Each segment was windowed with a Hamming window. The periodograms of each segment were then averaged to obtain an estimate of the power spectral density of the signal. In addition to computing band powers, we calculated the spectral power ratio of *Theta*_*frontal*_*/Beta*_*frontal*_^47–49^ at the Fz electrode.

With respect to ERP analysis, we focused on the P300 potential^52,56,57^. We applied an oddball paradigm before and after the experimental sessions (videoconference vs. face-to-face conditions). Specifically, we used the target category “faces” from the Affectnet dataset^79^ and “landscapes” from the Nencki Affective Pictures System (NAPS) dataset^80^. The probability of a target event (i.e., presentation of a face) was 10% (50 out of 500 events for each participant). Each image presentation lasted 500 ms. The oddball paradigm can be found in the Github repository of one of the authors of the present paper, see https://github.com/scwLab. EEG data was band-pass filtered (two-way least-squares FIR filtering) between 0.5 and 30 Hz, followed by epoching into trials specific to the target event. We removed outlier trials using amplitude thresholding, specifically by discarding trials where EEG amplitude exceeded ± 100 μV, and computed the grand average ERPs across all participants in Fz, Cz, Pz, O1, and O2 channels.

We also utilized ECG data to identify potential fatigue effects of the 50-min lecture by analyzing HR and computing various HRV indices in non-overlapping 3-min windows. We evaluated time-domain measures by analyzing the HR signal (heartbeat intervals RR) extracted from the ECG using the Pan-Tompkins algorithm^81,82^ and resampled to 4 Hz. Specifically, we considered the following measures:

- HR (bpm): Mean heart rate in beats per minute.
- RMSSD (ms): The root mean square of successive differences between normal heartbeats^67^. RMSSD describes the variance of the beat-to-beat intervals and reflects short-term rapid changes in heart rate due to parasympathetic modulation^83^.
- pNN50 (%): Proportion of NN50 divided by the total number of RR intervals. NN50 is the number of times in which successive heartbeat intervals exceed 50 ms^84^. Similarly to RMSSD, pNN50% captures vagally mediated HRV changes^83^.
- SDRR (ms): Standard deviation of the RR intervals over the observed period of time^67^.

We also extracted frequency-domain measures by applying spectral analysis on the heart rate signal. Welch’s method^85^ allowed us to compute the heart rate’s power spectrum from which we estimated the following indices:

- HF_nu_: Normalized high frequency (HF; 0.15–0.4 Hz) power, defined as HF power divided by the total power up to 0.4 Hz. HF_nu_ describes the relative contribution of the high frequencies to the total power of the heart rate signal and is usually interpreted as an index of vagal tone^67^.
- LF_nu_: Normalized low frequency (LF; 0.04–0.15 Hz) power, defined as LF power divided by the total power up to 0.4 Hz. LF_nu_ represents the relative contribution of the low frequencies to the total power of the heart rate signal and is usually linked with the sympathetic modulation of the ANS^67^.
- LF/HF: The LF power divided by the HF power. The LF/HF ratio is commonly used as a measure of sympathovagale balance; in the present paper we use the natural logarithm of the LF/HF ratio, ln[LF/HF]^67^.

Table 1 summarizes all measures used in the present study to determine fatigue.

**Table 1:** Fatigue measures of the present study.

Tool and method	Fatigue measure	Major sources
Continuous EEG	Increases in Theta and Alpha activities	^42,43^
Ratios between different EEG frequency bands	Increased ratio of *Theta*_*frontal*_*/Beta*_*frontal*_	^45–49,86^
Event-Related Potential (EEG)	Changes in P300, P3a, and N2 components (based on oddball paradigm)	^56,57,59,62^
Electrocardiography (ECG)	Decreased HR	^30–32^
Electrocardiography (ECG)	Increased HRV (based on RMSSD, pNN50, SDRR, HF_nu_, LF_nu_, and ln[LF/HF])	^30–32^
Self-report measures	Brief Mood Introspection Scale (BMIS) and Zoom Exhaustion and Fatigue (ZEF) Scale	^11,75^

The reliability of the BMIS and ZEF questionnaires was evaluated using Cronbach’s alpha. Additionally, these self-report responses obtained during both the videoconferencing and face-to-face sessions were compared using the Wilcoxon signed rank test. To address the issue of multiple comparisons we employed the Benjamini–Hochberg method^87^.

In the oddball paradigm, we utilized linear mixed effects (LME) models^88^ to assess the impact of the lecture format (videoconferencing, face-to-face) on the recorded ERP signals during target events (i.e., faces). For each lecture format and each time sample, the ERP signal amplitude (response variable) was modeled as a linear combination of fixed effects and random effects. Specifically, we used an ordinal vector as the fixed effect predictor to indicate if the oddball was presented before or after the lecture (*Phase*) and included a by-subject random intercept (1|*Participant*) to control for inter-subject variability,

1 $$ERP\left(n\right) \sim Phase+\left(1|Participant\right)$$

where $ERP\left(n\right)$ refers to the ERP amplitude at time point $n$. The fixed effect *p*-values were then used to identify statistically significant ERP amplitude changes related to lecture format. To investigate potential links between BMIS items and changes in the ERP signals we applied again LME models. We used the ERP signal amplitude after the lecture (${ERP}_{after}$) as the response variable and considered the ERP amplitude before the lecture (${ERP}_{before}$) and BMIS item scores before and after the lecture (${{BMIS}^{(i)}}_{before}$ and ${{BMIS}^{(i)}}_{after}$) as fixed effects. A by-subject random intercept was also included^89^:

2 $${ERP}_{after}\left(n\right) \sim {ERP}_{before}\left(n\right) +{{BMIS}^{(i)}}_{before}+{{BMIS}^{(i)}}_{after} +\left(1|Participant\right)$$

where $(i)$ refers to the i^th^ item of the BMIS questionnaire. For the ZEF questionnaire, we applied the same methodology using, however, only ZEF item scores obtained after the lecture as fixed effect predictors,

3 $${ERP}_{after}\left(n\right) \sim {ERP}_{before}\left(n\right) +{{ZEF}^{(i)}}_{after} +\left(1|Participant\right)$$

where $(i)$ refers to the i^th^ item of the ZEF questionnaire. Prior to these calculations, each item was normalized to zero mean and unit standard deviation (among all subjects and conditions). To quantify the magnitude and the directionality (i.e., sign) of the associations between different BMIS or ZEF items and the resulting ERP amplitude, we estimated the fixed effects of the scores after the lecture (i.e., ${{BMIS}^{(i)}}_{after}$ or ${{ZEF}^{(i)}}_{after}$) and standardized them by the standard deviation of the model residuals. Their *p*-values, corrected for multiple comparisons, were used to assess the statistical significance of the effect in time. The primary aim of this procedure was to eliminate any potential regression to the mean effects^90^ and monitor the relationship between the reported mood/fatigue levels and the time-varying changes in the ERP signals after each lecture.

In the continuous EEG and ECG analysis, our primary objective was to observe changes in EEG band powers and HRV indices over time using non-overlapping 3-min intervals throughout the entire lecture. To evaluate significant differences between the videoconferencing and face-to-face sessions, we employed the Wilcoxon signed rank test, considering both temporal and average (over time) variations. Correction for multiple comparisons (e.g., time, channels) was achieved through the Benjamini–Hochberg method.

Our subsequent objective was to establish a deeper understanding of the interplay between physiological and self-reported measures, specifically investigating whether the identified physiological differences were correlated with the participants’ self-reported mood and fatigue levels. As in the oddball, the relationship between different BMIS or ZEF items and the average EEG and ECG indices over the whole duration of the class was evaluated using LME models. The average value of an EEG and ECG index (${Index}_{average}$) was assigned as the response variable and the after and/or before lecture scores as fixed effect factors,

4 $${Index}_{average}={{BMIS}^{(i)}}_{before}+{{BMIS}^{(i)}}_{after} +\left(1|Participant\right)$$

5 $${Index}_{average}={{ZEF}^{(i)}}_{after} +\left(1|Participant\right)$$

The standardized fixed effect of ${{BMIS}^{(i)}}_{after}$ or ${{ZEF}^{(i)}}_{after}$ was then used to quantify the relationship between EEG and ECG indices and the BMIS and ZEF items scores provided after the lecture controlling for the effect of the scores before the lecture. Eq-(4) and Eq-(5) were estimated using data from all participants and both lecture types.

### Ethics declaration

The study was conducted according to the Declaration of Helsinki, participants gave written informed consent, and the study protocol was approved by the ethical review board of Graz University of Technology.

## Results

### BMIS and ZEF questionnaires

**Figure 2:**
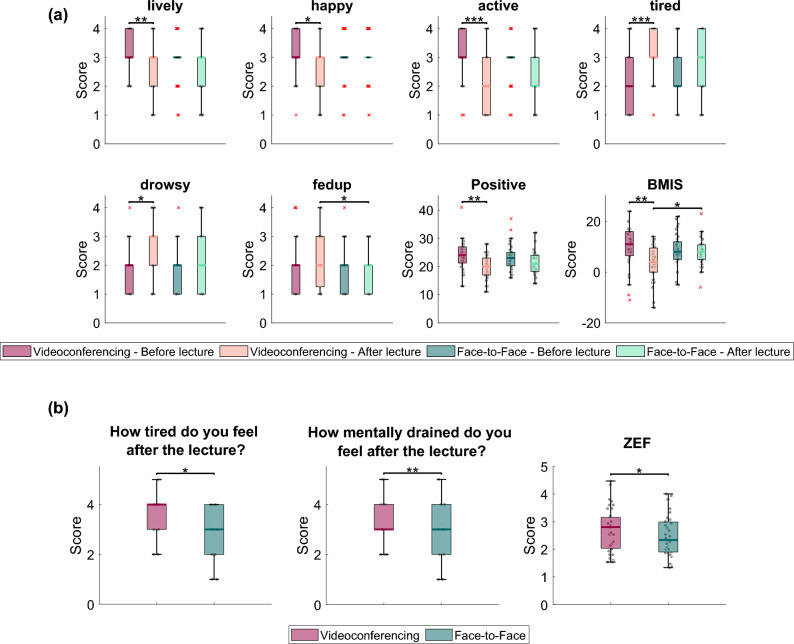
(**a**) Boxplots depicting the distribution of the BMIS adjective scores before and after each lecture (as denoted in the legend). Positive score refers to the total score of the positive mood adjectives. BMIS refers to the BMIS overall score (see Supplementary Material for more information). (**b**) Boxplots depicting the distribution of ZEF item scores (as denoted in the legend) after each lecture. The first two questions belong to the General Fatigue category. ZEF refers to the overall ZEF score defined as the average score over all fatigue items (see Supplementary Material for more information). Statistically significant differences between conditions are indicated with asterisks (*p < 0.05, **p < 0.01, ***p < 0.001).

The BMIS and ZEF questionnaires showed high reliability in the present sample, as indicated by the Cronbach’s alpha (> 0.7, see Supplementary Material for details). Figure 2 shows the descriptive statistics and *p*-values for the statistically significant BMIS (Fig. 2a) and ZEF (Fig. 2b) items, respectively.

Analysis of the BMIS questionnaire responses revealed that the participants felt significantly more tired, drowsy, and fed up as a consequence of participation in the videoconferencing session, if compared to participation in the face-to-face session; moreover, they also felt less lively, happy, and active. Overall, the positive score (i.e., the composite measure across all positive adjectives) and the BMIS score in general were significantly lower in the videoconferencing condition compared to the face-to-face condition. General fatigue items (as a part of the ZEF score) and the ZEF score itself exhibited also significantly higher values as a consequence of participation in the videoconferencing session, if compared to participation in the face-to-face session.

In essence, what follows based on our self-report results is that participation in the videoconferencing session resulted in higher fatigue perceptions than participation in the face-to-face session. Note that we applied an LME model to check the impact of the order of the lectures (as well as its interaction with the type of the lecture) on the BMIS and ZEF scores and we found no significant effect.

### Continuous EEG

**Figure 3:**
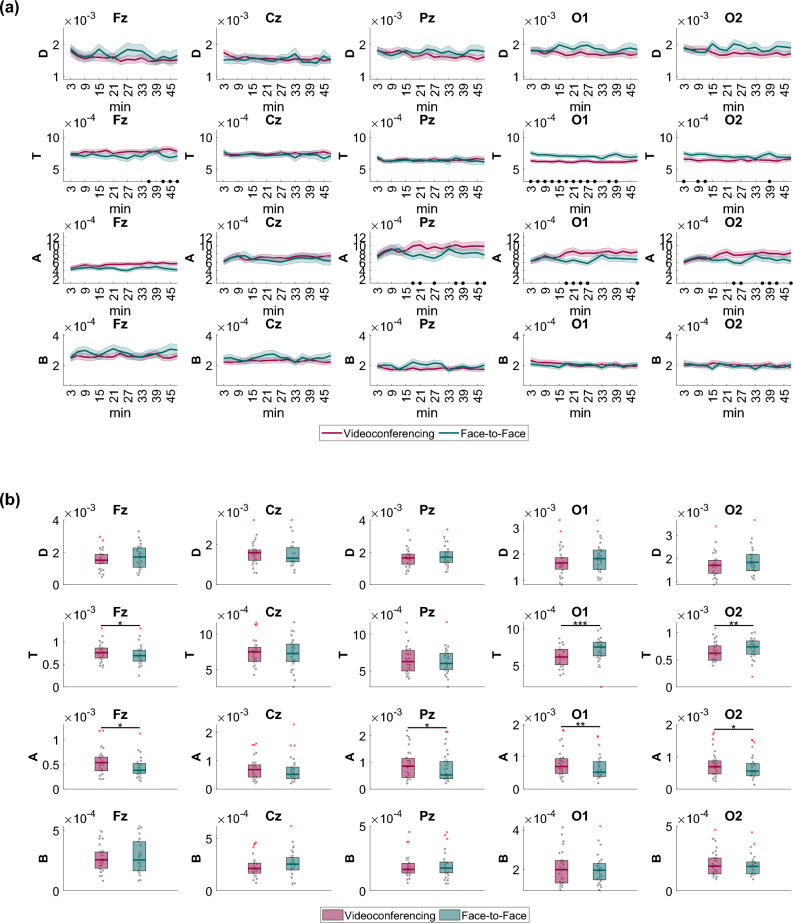
(**a**) Temporal evolution of the average (± standard error) of the EEG band power in the Delta (D; first row), Theta (T; second row), Alpha (A; third row), and Beta (B; fourth row) frequency bands during the videoconferencing (red lines) and face-to-face sessions (green lines). Each column represents a different EEG channel (Fz, Cz, Pz, O1, and O2 from left to right). Statistically significant differences (*p* < 0.05) between the two conditions are denoted with black dots on the temporal axis of each subplot. (**b**) Boxplots illustrating the distribution of the average over time EEG band power (Delta (D): first row, Theta (T): second row, Alpha (A): third row, Beta (B): fourth row) within participants for the videoconferencing (red) and the face-to-face (green) conditions. Each column represents different EEG channels (Fz, Cz, Pz, O1, and O2 from left to right). Statistically significant differences between the two conditions are denoted with asterisks (*p < 0.05, **p < 0.01, ***p < 0.001).

EEG band power values in the Delta (D), Theta (T), Alpha (A), and Beta (B) bands were extracted from 3-min non-overlapping windows. The time course of the band powers across all five EEG channels (Fz, Cz, Pz, O1, O2) is presented in Fig. 3a, providing a comprehensive overview of their temporal fluctuations. We also conducted linear regression analysis, fitting a line to the average data from each condition. Subsequently, we assessed whether the slope of the line exhibited statistical significance, indicating either a positive or negative trend with time. Furthermore, Fig. 3b shows boxplots that represent the distribution of the average band power values throughout the entire lecture duration. This provides a summary of the overall levels of the EEG power in both experimental conditions. Significant differences were detected in the Theta band (hereafter T Band) at frontal and occipital channels and in the Alpha band (hereafter A band) at parietal and occipital channels. Specifically, we observed the following results:

- T power was larger in Fz during videoconferencing, exhibiting a steady and slight increase throughout the lecture (slope = 2.7240e-06, *p* = 0.0145). However, in the face-to-face condition, T power showed fluctuations around a constant level. The T power differences between the videoconferencing and face-to-face conditions were significant during the last 15 min of the lecture.
- T power exhibited higher levels in O1 and O2 during the face-to-face condition compared to the videoconferencing condition.
- A power was more pronounced in Fz, Pz, O1, and O2 during the videoconferencing condition compared to the face-to-face condition. In the videoconferencing condition, we also observed a slight increase of A power over time in all channels (Fz channel: slope = 5.3551e-06, *p* = 0.0004, Cz channel: slope = 4.8269–06, *p* = 0.0107, Pz channel: slope = 1.0847e-05, *p* = 0.0012, O1 channel: slope = 1.2191e-05, *p* = 0.0002, O2 channel: slope = 1.2820e-05, *p* = 4.7520e-05).

**Figure 4:**
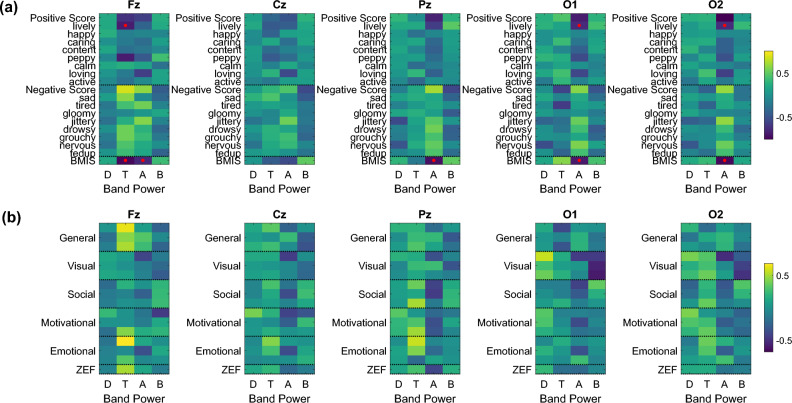
Relationship between band power and BMIS and ZEF items. Standardized fixed effects of (**a**) BMIS and (**b**) ZEF item scores on Fz (first column), Cz (second column), Pz (third column), O1 (fourth column), and O2 (fifth column) band power values. The y-axis of each subplot contains questionnaire items, while the x-axis refers to the power in different frequency bands (i.e., Delta (D), Theta (T), Alpha (A), and Beta (B)). The sign of the effect represents the directionality of the association. A yellow color gradient indicates a positive relationship, while a blue color gradient indicates a negative relationship. Red dots denote statistically significant associations (*p* < 0.05). Note that each ZEF dimension contains three items as described in the Supplementary Material (e.g., general fatigue contains three questions that have been merged to one dimension in this figure and the same applies for visual, social, motivational, and emotional fatigue).

The relationships between band power and BMIS and ZEF items are summarized in Fig. 4. Significant relationships were detected in frontal, parietal, and occipital channels in the T and A bands. Increases in frontal T and A band power values led to decreases in the BMIS score and specifically feelings of liveliness (Fig. 4a). The same applies for parietal and occipital A power (Fig. 4a). ZEF items did not provide significant correlations, however frontal T power was linked to higher general and emotional fatigue scores (Fig. 4b).

**Figure 5:**
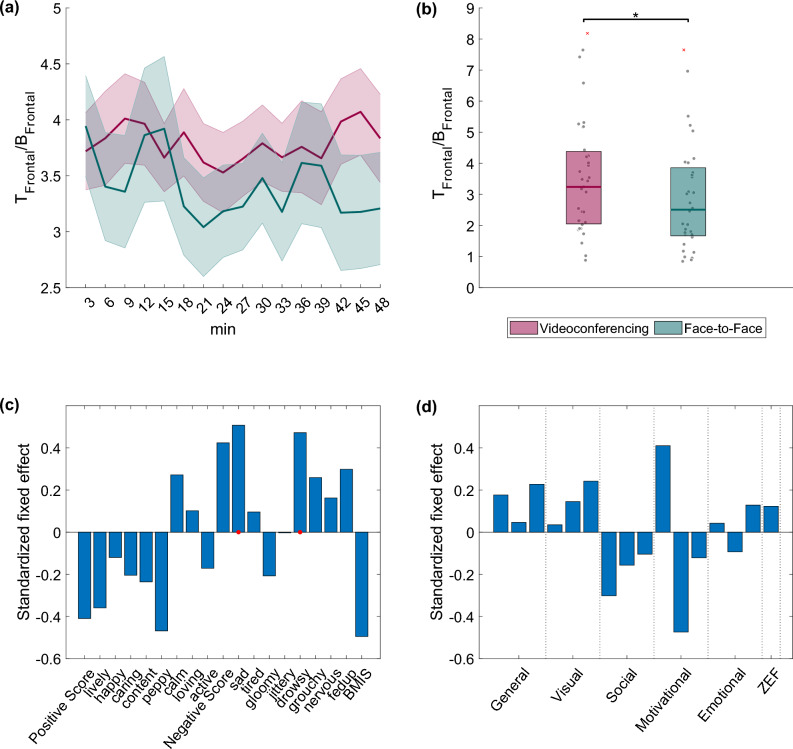
(**a**) Temporal evolution of the *Theta*_*frontal*_*/Beta*_*frontal*_ ratio during the videoconferencing (red) and face-to-face (green) conditions (no statistically significant differences between the conditions were found over time), along with (**b**) boxplots (right panel) depicting the average over time *Theta*_*frontal*_/*Beta*_*frontal*_ ratios within participants. Statistically significant differences between the conditions are denoted with asterisks (*p < 0.05). (**c**) Relationship between *Theta*_*frontal*_*/Beta*_*frontal*_ and BMIS items (standardized fixed effects of BMIS item scores on *Theta*_*frontal*_*/Beta*_*frontal*_). (**d**) Relationship between *Theta*_*frontal*_*/Beta*_*frontal*_ and ZEF item scores (standardized fixed effects of ZEF items scores on *Theta*_*frontal*_*/Beta*_*frontal*_). Red dots on the x-axis of each subplot denote statistically significant associations (*p* < 0.05). Positive (negative) fixed effects indicate a positive (negative) correlation between scores and band power ratios.

Statistically significant distinctions in the *Theta*_*frontal*_*/Beta*_*frontal*_ ratio, over time, were not observed between the videoconferencing and face-to-face sessions (Fig. 5a). Nonetheless, it is noteworthy that, overall, the *Theta*_*frontal*_*/Beta*_*frontal*_ exhibited a significantly greater value in the videoconferencing session when contrasted with the face-to-face session (Fig. 5b). Higher *Theta*_*frontal*_*/Beta*_*frontal*_ ratios were also found to be positively associated with feelings of sadness and drowsiness (Fig. 5c). ZEF items did not provide significant correlations (Fig. 5d).

### Event-related potential (ERP) (oddball task)

**Figure 6:**
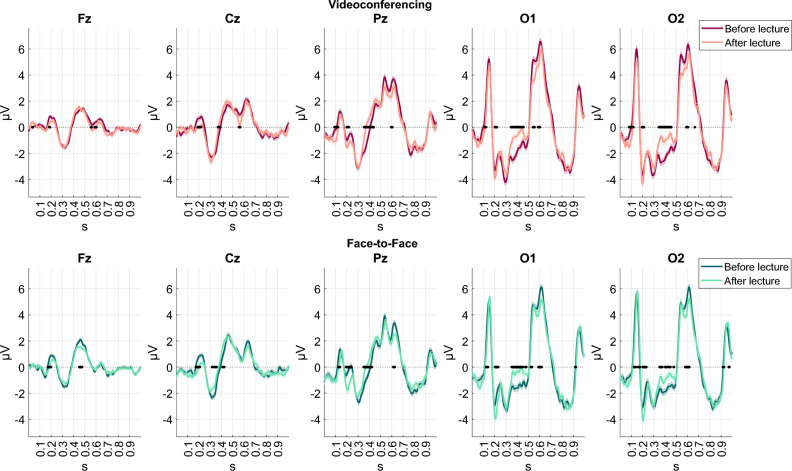
Grand-average ERP signals during a target event (i.e., faces) of the oddball paradigm before and after the videoconferencing (top panel) and the face-to-face (bottom panel) conditions. Columns represent different channels (Fz, Cz, Pz, O1, and O2 from left to right). Statistically significant ERP changes (*p* < 0.05) after each condition are denoted with black dots on the x-axis of each subplot.

In Fig. 6, we present the grand average Fz, Cz, Pz, O1, and O2 ERPs elicited from target stimuli of the oddball task before and after the videoconferencing and face-to-face conditions. Statistically significant ERP changes after each condition are denoted with black dots on the x-axis of each plot. The effect of experimental condition (videoconferencing, face-to-face) was most prominent on occipital channels and specifically on the onset latency and waveform amplitude of the P1 component, indicating some level of fatigue after both conditions. Herein, onset latency is defined as the time point at which the component waveform starts to increase from a baseline of zero and waveform amplitude as the amplitude of the positive or negative deflection of the corresponding component. No lateralized occipital activation was observed. We also detected significant ERP changes as a function of experimental condition on parietal, central, and frontal channels. Specifically, we found:

- A smaller Cz P3a onset latency after the videoconferencing condition and a slight nonsignificant increase in the waveform amplitude of the Cz N2.
- A smaller Cz P3a onset latency after the face-to-face session, and a significant drop in the Fz P3a and Cz N2 waveform amplitude.

To further analyze these changes, for each condition we combined all the subject-average ERP signals from all channels and applied LME models. The ERP amplitude at each time point was modeled as a linear combination of fixed effects and random effects, including the BMIS or ZEF scores before and after the corresponding condition as fixed effects and a by-subject random intercept to account for inter-subject variability (as described earlier in the Methods and Materials section). This approach allowed us to examine the relationship between the self-reported mood and fatigue levels and the changes in ERP signals after each condition.

**Figure 7:**
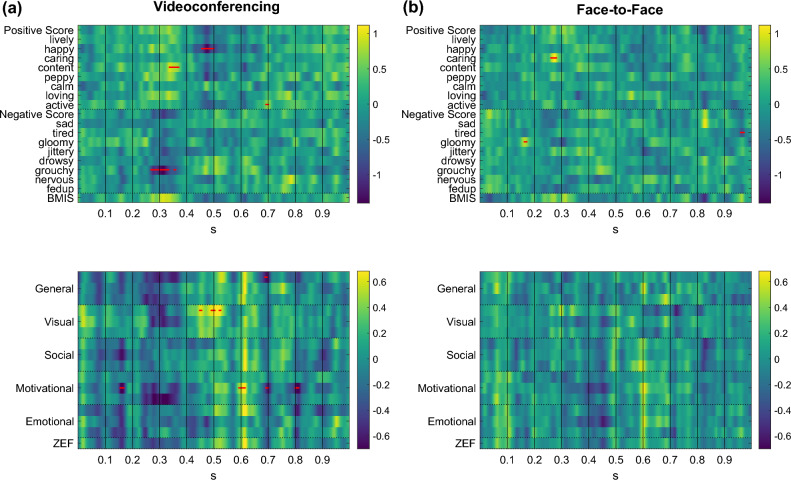
Time-varying standardized fixed effects of BMIS (top panel) and ZEF (bottom panel) item scores on the ERP amplitude recorded during the oddball paradigm after the (**a**) videoconferencing (left panel) and (**b**) face-to-face conditions (right panel). A yellow color gradient indicates a positive relationship, while a blue color gradient indicates a negative relationship. Red dots denote statistically significant associations (*p* < 0.05). These images describe the time-varying correlation of the ERP signal at each time point with the self-reported BMIS and ZEF item scores after the lecture controlling for the effect of the corresponding scores before the lecture.

Specifically, our analysis showed that the videoconferencing condition induced significant patterns. We observed a positive relationship between the waveform amplitude of the N2 component (~ 0.3 s) and the positive BMIS scale, as well as a negative relationship with the negative BMIS scale (Fig. 7a, upper panel). This suggests that increases in the waveform amplitude of the N2 (i.e., more negative values) were associated with negative feelings (especially “grouchy”). Similarly, an increase in the waveform amplitude of the P3a (between 0.4 and 0.5 s) was linked to a lower score of positive feelings (especially “happy”). This trend was also reflected in the ZEF survey items, whereby larger N2 and P3a waveform amplitudes resulted in higher fatigue scores (Fig. 7a, bottom panel). Notably, we found a significant positive correlation between P3a and visual fatigue. Significant positive associations related to the P300 were also observed between 0.5 s and 0.7 s. These associations were more pronounced in the videoconferencing condition compared to the face-to-face condition. For the face-to-face condition no significant patterns were detected (Fig. 7b).

Overall, these findings indicate that the ERP changes after the videoconferencing condition were more strongly associated with mental fatigue and negative feelings compared to the face-to-face condition. The N2 and P3a components were the most important components related to these changes.

### ECG analysis

**Figure 8:**
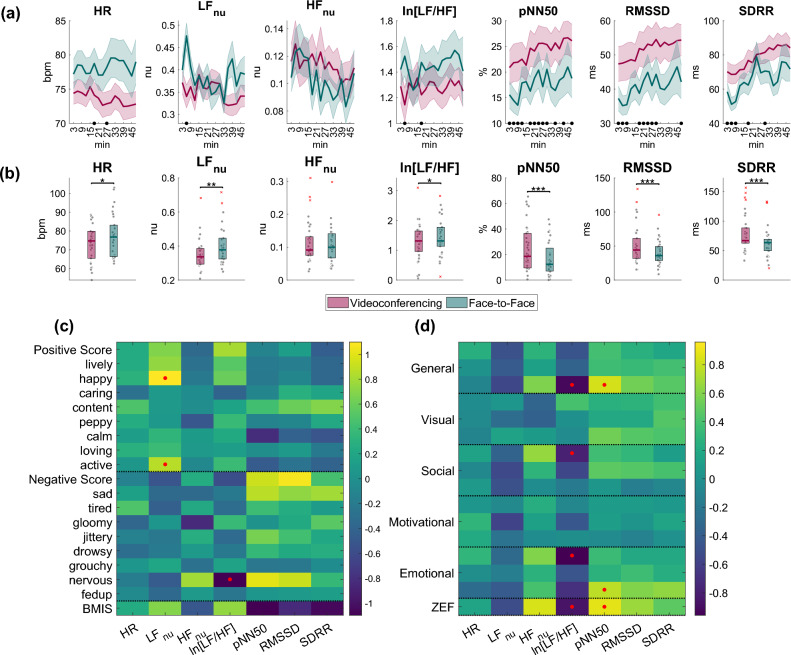
(**a**) Temporal progression of HRV indices during the videoconferencing (red) and face-to-face conditions (green). (**b**) Boxplots of the average over time indices (within participants) for both conditions. Statistically significant differences between the conditions are denoted with asterisks (*p < 0.05, **p < 0.01, ***p < 0.001). (**c**) Relationship between ECG features and BMIS items (standardized fixed effects of BMIS item scores on various HRV indices (i.e., HR, LF_nu_, HF_nu_, ln[LF/HF], pNN50, RMSSD, and SDRR)). (d) Relationship between ECG features and ZEF items (standardized fixed effects of ZEF item scores on HRV indices). The sign of the effect represents the directionality of the association. A yellow color gradient indicates a positive relationship, while a blue color gradient indicates a negative relationship. Red dots denote statistically significant associations (*p* < 0.05). Note that each ZEF dimension contains three items as described in the Supplementary Material (e.g., general fatigue contains three questions that have been merged to one dimension in this figure and the same applies for visual, social, motivational, and emotional fatigue).

HR, as well as HRV features, were continuously monitored during the entire duration of the lecture using 3-min non-overlapping windows. Figure 8a illustrates the temporal progression of HR, LF_nu_, HF_nu_, ln[LF/HF], pNN50, RMSSD, and SDRR during the videoconferencing and the face-to-face conditions. We applied linear regression analysis and fitted a line to the average data from each condition. We then determined whether the slope of the line was significantly positive or negative, indicating a positive or negative trend with time. Specifically, we observed the following:

- A gradual decline in HR during the videoconferencing session (slope = -0.1482, *p* = 0.0003).
- A reduction in HF_nu_ in both conditions over time (videoconferencing: slope = -0.0011, *p* = 0.0027; face-to-face: slope = -0.0017, *p* = 0.0086).
- A gradual increase in pNN50 (videoconferencing: slope = 0.3572, *p* = 7.1075e-06; face-to-face: slope = 0.2828, *p* = 0.0085), RMSSD (videoconferencing: slope = 0.4955, *p* = 2.0640e-06; face-to-face: slope = 0.4773, *p* = 0.0022), and SDRR (videoconferencing: slope = 1.3040, *p* = 1.0294e-08; face-to-face: slope = 1.2021, *p* = 0.0017) in both conditions.

We also compared the average over time indices (within participants) for both conditions (Fig. 8b). Overall, participants in the face-to-face condition exhibited significantly higher HR, LF_nu_, and ln[LF/HF]. However, the participants in the videoconferencing session exhibited significantly higher pNN50, RMSSD, and SDRR.

Through analyzing the HRV indices alongside the results of the BMIS and ZEF questionnaires, we identified significant associations between the level of these HRV indices and the participants’ mood and fatigue self-reports. In Fig. 8c,d, we provide standardized fixed effect matrices that illustrate the positive or negative association between each index and each item of the BMIS and ZEF questionnaires. Regarding BMIS, significant associations were detected between LF_nu_ and active and happy (positive relationship) and ln[LF/HF] and nervous (negative relationship). However, correlation patterns were not as distinct as in the ZEF. For example, pNN50, RMSSD, and SDRR were mostly positively linked to negative feelings, but to some extent also to pleasant feelings. We also found this pattern for other indices, including HR, HF_nu_, and ln[LF/HF]. Regarding ZEF, the *p*-values revealed that general fatigue, emotional fatigue, and ZEF were significantly positively associated with pNN50 (and also with RMSSD and SDRR, but not significantly), while ln[LF/HF] was significantly negatively correlated with general fatigue, social fatigue, emotional fatigue, and the ZEF score.

## Discussion

Self-report findings in the extant literature show that people often perceive elevated fatigue as a consequence of participation in videoconferences^12–16^. Our self-report results confirm this finding. Our participants reported to feel significantly more fatigued, tired, drowsy, and fed up, as well as less lively, happy, and active, as a consequence of participation in the videoconferencing condition, if compared to participation in the face-to-face condition. Also, mood in general worsened as a consequence of participation in the videoconferencing condition compared to the face-to-face condition. Importantly, the major contribution of the present study to the academic literature is that it complements self-report measurement with neurophysiological measurement, specifically with EEG and ECG data. Our findings show that videoconferencing also comes along with fatigue on a neurophysiological level.

We assessed users’ ongoing EEG during videoconferencing and analyzed the ERP components before and after videoconferencing based on a cognitive attention task (i.e., oddball paradigm). We contrasted the results with a face-to-face condition. In essence, we identified notable differences between the videoconferencing and face-to-face conditions, showing that videoconferencing fatigues the brain more than the face-to-face interaction. To the best of our knowledge, this finding is the first report in the academic literature indicating that use of videoconferencing tools may lead to fatigue on a brain level based on EEG data.

Regarding the ongoing EEG, our results show increased frontal Theta power during the videoconferencing session, indicating brain fatigue^42,43^. Moreover, this increased frontal Theta power was linked to lower levels of liveliness (a component of the BMIS score) and the overall BMIS score, as well as to increased general fatigue at trend level (a component of the ZEF score), if compared to the face-to-face condition. Mentally fatigued individuals may have to exert higher levels of effort in order to maintain attention and this could explain higher frontal Theta^40,91^. Frontal, parietal, and occipital Alpha power was also linked to a lower overall BMIS score. Earlier research has established a connection between Alpha suppression and task engagement and alertness^92^. Since the parietal and occipital Alpha power levels were lower during the face-to-face condition, this could indicate higher levels of engagement in the face-to-face condition. Interestingly, Theta power was significantly higher in the occipital channels in the face-to-face condition if compared to the videoconferencing condition. It is possible that this increased occipital Theta power is caused by heightened visual processing; for example, due to the observation of nonverbal communication cues like the lecturer’s facial expressions. Empirical evidence substantiates the importance of increased Theta occipital oscillations in the recognition of emotional facial expressions^93^.

Regarding ratios between different EEG frequency bands, the *Theta*_*frontal*_*/Beta*_*frontal*_ ratio exhibited significant correlations with negative feelings, in particular drowsiness and sadness (positive correlation). Considering that research found that increased *Theta*_*frontal*_*/Beta*_*frontal*_, among others, signifies impaired attention control^47,48^ and episodes of mind-wandering if directly compared to focused attention^49^, it follows that videoconferencing reduces cognitive attention, a relationship which is presumably mediated by increased fatigue.

Regarding ERP analysis based on the oddball paradigm, the changes observed in the N2 and P3a components after videoconferencing suggest that individuals experiencing greater fatigue may have reduced attentional resources leading to faster P3a responses and slightly higher waveform amplitudes in the N2 component. Fatigue (and especially visual fatigue) may have a greater impact on attention and cognitive processing in the frontal regions of the brain during online lectures. Increases in task difficulty also could increase frontal attentional focus to maintain task performance^59,60,94^. Furthermore, there has been evidence of stress-related enhancements in the N2 peak amplitude^61,95^, as well as fatigue induced modulations due to impaired cognitive control and altered cardiac autonomic regulation^62^. In contrast, the decreased frontal P3a observed after the face-to-face lecture does not appear to have a significant after-effect concerning mood or fatigue on a self-report level (as indicated by the BMIS and ZEF scores, see Fig. 7). It is also possible that this decrease could indicate faster habituation with the oddball paradigm in the face-to-face condition if compared to the videoconferencing condition^96^. This rationale is based on the fact that throughout human history the majority of interaction among people has taken place in face-to-face settings. Thus, unlike human interaction via videoconferencing tools, perception of a human face in a face-to-face setting and related cognitive mechanisms (such as habituation) must be part of the genetic makeup of humans^9^. However, future research is necessary to empirically confirm different interpretations of our results.

With respect to ECG data, we found that the strongest predictors of fatigue are the HRV measures pNN50, RMSSD, and ln[LF/HF]. The progressive increase of pNN50 and RMSSD with ongoing time in both conditions (videoconference, face-to-face) suggests a gradual increase in general fatigue throughout the lecture. This trend is substantiated by the ZEF scores. This observation is in line with the hypothesis that mental fatigue leads to enhanced vagal tone^30–32^. The face-to-face environment, in general, was associated with lower levels of pNN50, RMSSD, and SDRR, indicating reduced mental strain. Additionally, the face-to-face environment was linked to increased ln[LF/HF] (Fig. 8b), suggesting a more positive mood among participants compared to the videoconferencing environment^66^.

The present study has limitations which could be addressed in future research. First, in our experiment we used a teaching context (because videoconferencing plays a significant role in this domain). However, it is recommended that future research replicates our findings in other contexts. Specifically, we recommend replication of our study in a business context to simulate remote business meetings; this context—just as our education context—is critical due to the increased trend towards home office^20^.

Second, our study was deliberately designed as laboratory experiment. The major implication of this design choice is that our subjects not only participated in the face-to-face condition in a typical classroom setting in the study, but also in the videoconferencing session. Thus, the videoconferencing session was conducted in a room at the university and hence participants were not in a classical remote setting (e.g., at home). This decision to run the study as a laboratory experiment, and not as field experiment, opens up future research potential. It was our goal to have full control over our neurophysiological measurement tools (EEG, ECG) to contribute to measurement quality and internal validity of our findings. However, future research could emphasize external validity and hence replication of our study could be conducted in a remote setting. Here, participants could be asked to participate in the videoconferencing condition at home, sitting in front on their computers. Considering that several consumer-grade EEG instruments are available and can provide acceptable measurement quality in specific research situations (for a review, see^97^), along with the fact that smartwatches make possible a relatively effortless collection of HR and HRV data in remote contexts^98^, future field studies are recommended in the current study domain.

Third, the mean age of our participants was 24.06 years with a low standard deviation of 2.04. We deliberately defined this narrow age range to hold age as a factor constant in the current study. However, because mental fatigue is related to age^99^ the current findings cannot be simply generalized to older age groups. Rather, future research with older age groups (e.g., people in their 50 s and 60 s) is necessary to replicate the current findings. Based on recent evidence^100^, however, our prediction would be that the fatigue potential of videoconferencing is even higher in older individuals. Future empirical research could test this prediction.

Fourth, in our study we applied a research approach based on a limited number of EEG channels (Fz, Cz, Pz, O1, O2). It follows that our approach does not allow for an analysis of brain mechanisms that is similar to analyses based on measurements with 32, 64, or 128 channels. Thus, future research should replicate our study based on a higher number of channels in order to make possible further insights into the neural correlates of videoconferencing.

Fifth and finally, the relatively small number of significant differences observed in certain comparisons between the face-to-face and videoconferencing conditions may be attributed to the sample size. While our sample size resembles that of comparable neurophysiological research^73,74^, future studies should consider the inclusion of a larger pool of participants.

Reports on videoconference fatigue, both in practice and science, suggest a number of countermeasures to reduce the fatigue and stress potential. However, scientific research has not yet started to systematically evaluate the efficacy of these countermeasures. Rather, currently these suggestions typically have the status of unproven ideas. As an example, evidence in fields like human–computer interaction has shown that 10 min breaks during interaction with digital technologies (e.g., desktop computer with application system) may effectively reduce user fatigue and stress^26^. Such knowledge could be used in future research to develop hypotheses to test them directly in the context of videoconferencing, but also has implications for the development of neuroadaptive systems^101^. Such systems could, based on real-time measurement of users’ fatigue levels, suggest break times based on notifications.

## Concluding statement

The worldwide videoconferencing market was estimated at 25 billion US dollars in 2022 and is projected to experience an annual growth rate of over 10% from 2023 to 2032, resulting in a market size of 65 billion US dollars in 2032; the primary driver behind this and similar market forecasts is the increasing trend of remote work and online learning^102^. Thus, videoconferencing as a means of communication in both business and society is expected to gain further significance in the future, building upon an already high level of adoption today^5^. Due to several findings from survey studies in recent years reporting on fatigue as a consequence of videoconferencing^12–16^, we conducted an experimental study to compare the fatigue effects of communication through a videoconferencing tool versus face-to-face communication. In our study, importantly, we employed both survey instruments *and* neurophysiological measurement methods to assess fatigue^12–16^. We collected and analyzed EEG (continuous and event-related) and ECG (HR and HRV) data. Our neurophysiological data—together with the self-report data—show that videoconferencing, if compared to a face-to-face condition, results in neurophysiological changes which, based on existing literature, indicate fatigue. Thus, our results suggest that use of videoconferencing may lead to cognitive costs, which must not be ignored by individuals and organizations. However, as it is unrealistic to recommend completely abstaining from the use of videoconferencing tools, the future study of effective countermeasures to reduce the fatigue and stress potential of videoconferencing will be critical for sustaining human well-being and health in an increasingly digital world.

### Supplementary Information

Supplementary Information.

## Data Availability

The datasets used during the current study are available from the authors on reasonable request.
