# Supplementary material for: Videoconference fatigue from a neurophysiological perspective: experimental evidence based on electroencephalography (EEG) and electrocardiography (ECG)
Source: Sci Rep. 2023 Oct 26;13:18371. doi: 10.1038/s41598-023-45374-y (PMC10603122; doi:10.1038/s41598-023-45374-y)
Supplement: Supplementary file 1 — Supplementary Information. [file 41598_2023_45374_MOESM1_ESM.docx]

**Supplementary Material**

**Videoconference Fatigue from a Neurophysiological Perspective: Experimental Evidence based on Electroencephalography (EEG) and Electrocardiography (ECG)**

René Riedl^1,2,~,*^, Kyriaki Kostoglou^3,~^, Selina C. Wriessnegger^3,4^, Gernot R. Müller-Putz^3,4^

^1^ University of Applied Sciences Upper Austria, Campus Steyr, Digital Business Institute, Austria

^2^ University of Linz, Institute of Business Informatics – Information Engineering, Austria

^3^ Graz University of Technology, Institute of Neural Engineering, Austria

^4^ BioTechMed Graz, Graz, Austria

~ Shared first authorship

*** Corresponding Author**

Prof. Dr. René Riedl

rene.riedl@fh-steyr.at

*Brief Mood Introspection Scale (BMIS)*

The pleasant and negative adjectives that were used to describe the emotional state of the participant can be found in table S1. The participants were instructed to grade how well each adjective described their present mood. The scale and the corresponding scores assigned to each adjective can be also found in table S1. The overall mood was graded on a scale between -10 (very unpleasant) to 10 (very pleasant). The BMIS score was obtained by subtracting the total (i.e., sum) score of the negative adjectives from the total score of the positive adjectives. The results of the questionnaire can be found in Figure S1 in the form of boxplots.

| **Positive adjectives** | **Negative adjectives** |  | **Scale** | **Score** |
| --- | --- | --- | --- | --- |
| Happy | Sad |  | definitely do not feel | 1 |
| Lively | Tired |  | do not feel | 2 |
| Caring | Jittery |  | slightly feel | 3 |
| Content | Drowsy |  | definitely feel | 4 |
| Peppy | Fed up |  |  | |
| Loving | Gloomy |  |  |  |
| Active | Grouchy |  |  |  |
| Calm | Nervous |  |  |  |

**Table S1:** BMIS adjectives used to describe the emotional state of the participant along with grading scale


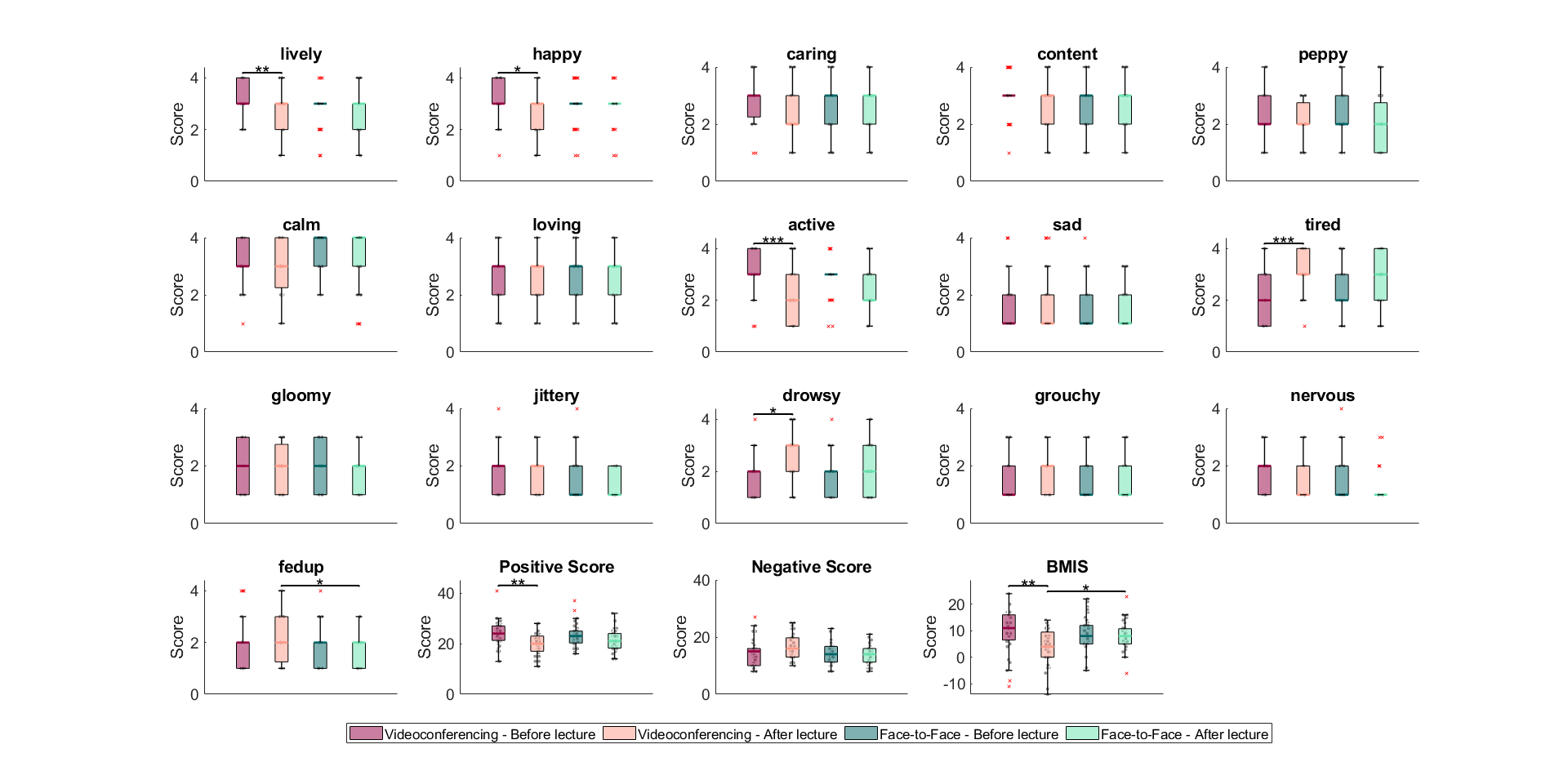


**Figure S1**: Boxplots depicting the distribution of the adjective scores before and after each lecture (as denoted in the legend). The last three boxplots refer to the total score of the positive (i.e., Positive), negative (i.e., Negative) adjectives, and the BMIS overall score.

*Zoom Exhaustion and Fatigue Scale (ZEF)*

The ZEF questionnaire was provided after the lecture in both the face-to-face and videoconferencing conditions. The ZEF is utilized for assessing various types of fatigue caused mainly by videoconferencing, including general, visual, social, motivational and emotional fatigue (https://stanfordvr.com/zef/) (see references 11 and 18 in the main manuscript). In this study, however, we also handed out the same questionnaire with some minor wording adjustments to the participants in the face-to-face condition for comparison purposes. Table S2 summarizes the survey questions. The minor adjustments referred to changing “after video conferencing” to “after the lecture”. As an example, the first item “How tired do you feel after video conferencing” was changed to “How tired do you feel after the lecture” in the face-to-face condition. All items indicated with an asterisk are measured on a scale from 1 to 5 as follows: 1 = *“Not at all”*, 2 = *“Slightly”*, 3 = *“Moderately”*, 4 = *“Very”*, 5 = *“Extremely”*). Questions with ID zef_16_ and zef_18_ range from 1 = *“Never”,* 2 = “*Rarely*”, 3 = “*Sometimes*”, 4 = “*Often*” to 5 = *“Always”*, and zef_17_  from 1 = *“Less than 15 min”*, 2 = *“15 to 30 min”*, 3 = *“30 to 45 min”*, 4 = *“45 min to an hour”*, 5 = *“More than an hour”*. The ZEF Score is computed as the averaged rating across all fatigue items except of zef_16_, zef_17_, and zef_18_. Figure S2 depicts the results of the survey.

| **Constructs** | **Questions** | **ID** |
| --- | --- | --- |
| General Fatigue | How tired do you feel after video conferencing? * | zef_3_ |
|  | How exhausted do you feel after video conferencing? * | zef_12_ |
|  | How mentally drained do you feel after video conferencing? * | zef_6_ |
| Visual Fatigue | How blurred does your vision get after video conferencing? * | zef_14_ |
|  | How irritated do your eyes feel after video conferencing? * | zef_9_ |
|  | How much do your eyes hurt after video conferencing? * | zef_15_ |
| Social Fatigue | How much do you tend to avoid social situations after video conferencing? * | zef_4_ |
|  | How much do you want to be alone after video conferencing? * | zef_8_ |
|  | How much do you need time by yourself after video conferencing? * | zef_13_ |
| Motivational Fatigue | How much do you dread having to do things after video conferencing? * | zef_2_ |
|  | How often do you feel like doing nothing after video conferencing? * | zef_10_ |
|  | How often do you feel too tired to do other things after video conferencing? * | zef_11_ |
| Emotional Fatigue | How emotionally drained do you feel after video conferencing? * | zef_7_ |
|  | How irritable do you feel after video conferencing? * | zef_5_ |
|  | How moody do you feel after video conferencing? * | zef_1_ |
|  | On a typical day, how many video conferences do you participate in? | zef_16_ |
|  | How long does a typical video conference last? | zef_17_ |
|  | On a typical day, how much time do you have between your video conferences? | zef_18_ |

**Table S2**: ZEF survey questions.

**Figure S2:** Boxplots depicting score distributions for all the ZEF items along with the ZEF score after each lecture (as denoted in the legend).

*Cronbach’s Alpha Reliability Coefficient*

The Cronbach’s alphas for the ZEF scores and for the positive and negative mood subscales of the BMIS can be found in Table S3. At first, we analyzed each lecture format separately before merging the scores obtained from both the videoconferencing and face-to-face conditions.

| **Lecture Type** | **Items** | **Cronbach’s alpha** |
| --- | --- | --- |
| Videoconferencing | BMIS Positive mood subscale | 0.733 |
|  | BMIS Negative mood subscale | 0.759 |
|  | ZEF | 0.897 |
| Face-to-Face | BMIS Positive mood subscale | 0.734 |
|  | BMIS Negative mood subscale | 0.725 |
|  | ZEF | 0.897 |
| Videoconferencing & Face-to-Face | BMIS Positive mood subscale | 0.732 |
|  | BMIS Negative mood subscale | 0.749 |
|  | ZEF | 0.897 |

**Table S3**: Cronbach’s alpha for ZEF and BMIS questionnaires
